# Using TBAg/PHA Ratio for Monitoring TB Treatment: A Prospective Multicenter Study

**DOI:** 10.3390/jcm11133780

**Published:** 2022-06-29

**Authors:** Xiaochen Wang, Mingwu Li, Guobiao Liu, Xiaoying Wu, Rong Wan, Hongyan Hou, Shiji Wu, Ziyong Sun, Haobin Kuang, Feng Wang

**Affiliations:** 1 Department of Laboratory Medicine, Tongji Hospital, Tongji Medical College, Huazhong University of Science and Technology, Wuhan 430030, China; wxcsingasong@163.com (X.W.); houhongyan89@163.com (H.H.); wilson547@163.com (S.W.); zysun@tjh.tjmu.edu.cn (Z.S.); 2 The Third People’s Hospital of Kunming, Kunming 650041, China; ynkmlmw@sina.com (M.L.); wanrong123@163.com (R.W.); 3 The First Institute of Guangzhou Tuberculosis Control Institute, Guangzhou Chest Hospital, Guangzhou 510095, China; liuguobiaodengho@sina.com; 4 The Second Branch of Guangzhou Chest Hospital, Guangzhou 510095, China; wuxiaoying86@tom.com; 5 Department of Tuberculosis, Guangzhou Chest Hospital, Guangzhou 510095, China

**Keywords:** tuberculosis, T-SPOT.TB, TBAg/PHA ratio, AUC, treatment outcomes

## Abstract

The way to monitor tuberculosis (TB) treatment is extremely lacking in clinical practice. The aim of the study is to assess the role of the TBAg/PHA ratio in the treatment monitoring of TB. TB patients were followed up for 6 months and serial T-SPOT.TB (T-SPOT) assays were performed. In patients with successful treatment outcomes, the ESAT-6 sfc, CFP-10 sfc, and TBAg/PHA ratio all showed a decreased trend after the initiation of treatment. Conversely, PHA sfc showed an increased trend after 2 months of treatment. However, these indicators had moderate performance in distinguishing between before and after 6 months of treatment, and the AUC ranged from 0.702 to 0.839. Notably, the TBAg/PHA ratio in patients without risk factors was of important value in differentiation between before and after treatment. The optimal AUC of TBAg/PHA ratio reached up to 0.890. Patients with unsuccessful treatment outcomes showed persistently high levels of TBAg/PHA ratio. The TBAg/PHA ratio in patients after 6 months of treatment showed a certain potential in distinguishing between patients with successful and unsuccessful treatment outcomes. A further calculation of the TBAg/PHA ratio in T-SPOT assay has potential value in the treatment monitoring of TB, but further confirmation is needed.

## 1. Introduction

Tuberculosis (TB) is one of the leading causes of death from a single infectious agent worldwide. Globally, an estimated 10.0 million people fell ill with TB in 2019, a number that has been declining slowly in recent years [1]. In addition, TB-related deaths dropped from 1.5 million in 2018 to 1.4 million in 2019 [1]. Although the TB incidence rate continually declined in the period from 2000 to 2019, the pace of progress worldwide is not yet fast enough.

The early diagnosis and effective treatment are still the key to end TB. In recent decades, the diagnosis of TB has greatly improved with the broad use of rapid molecular tests, whereas the methods for monitoring TB treatment are almost stagnant. Acid fast stain (AFS) and culture are still the commonly used methods for assessing the outcomes of TB treatment. Nevertheless, these two methods are not suitable for this purpose because of their low sensitivity or long turnaround time. The two commercially available interferon-gamma release assays, T-SPOT.TB (T-SPOT) and QuantiFERON-TB, have been proven to be useful to accurately detect *Mycobacterium tuberculosis* (Mtb) infection. However, these assays have limited usefulness for monitoring TB therapy [2]. Recently, some gene biomarkers proved to have potential value in monitoring TB treatment [3,4], but further validation studies are needed to confirm the role of these biomarkers. Due to the lack of effective ways to monitor TB treatment, both undertreatment and overtreatment are potentially serious in clinical practice.

T-SPOT assay is based on the detection of secretion of interferon-gamma by lymphocytes exposed to Mtb-specific antigens (TBAg). Previously, we have put forward that phytohemagglutinin (PHA) value in T-SPOT assay can reflect the immune status of the host. In addition, calculation of the ratio of TBAg to PHA (TBAg/PHA), a new application of T-SPOT assay, can eliminate the impact of individual immune variations on T-SPOT assay. Accordingly, the TBAg/PHA ratio had better performance than TBAg value in diagnosing active TB [5,6,7,8,9]. Considering that the abundance of Mtb-specific lymphocytes decreased with the decrease of bacterial burden after treatment, whereas the impaired lymphocyte function gradually restored without the persistent Mtb antigen exposure, we hypothesized that the decreased trend would be more pronounced for the TBAg/PHA ratio than TBAg value during TB treatment. In this study, we confirmed that the TBAg/PHA ratio in T-SPOT assay may be useful to monitor TB therapy.

## 2. Materials and Methods

### 2.1. Study Subjects

This is a prospective study which was performed in three tertiary hospitals (Tongji Hospital, a large teaching hospital located in the central region of China; Guangzhou Chest Hospital, a TB-specialized hospital located in the south of China; Kunming Third People’s Hospital, a TB-specialized hospital located in the west of China). The suspected pulmonary TB patients who had positive sputum AFS results were continuously recruited in the above three hospitals between July 2018 and September 2018. Mtb culture was performed with the mycobacteria growth indicator tube 960 system (Becton Dickinson, Sparks, MD, USA) and Lowenstein-Jensen media by using the same sputum specimens. Growth on either one of the two media was considered culture-positive. Mycobacterial species were identified using MPT 64 antigen detection and a drug susceptibility test was also performed. Patients diagnosed as non-tuberculous mycobacteria (NTM) infection, younger than 16 years of age, and those undergoing TB treatment were excluded from the study. Clinical information and conventional test results were collected from electronic patient records. This study was approved by the ethical committee of Tongji Hospital, Tongji Medical College, Huazhong University of Science and Technology (ID: TJ-IRB20190421); the ethical committee of Guangzhou Chest Hospital (ID: IRB201830); and the ethical committee of Kunming Third People’s Hospital (ID: IRB2019031917), Kunming, China.

### 2.2. Clinical and Follow-Up Procedures

The newly diagnosed TB patients received standard TB treatment, consisting of a daily four-drug combination (isoniazid, rifampicin, ethambutol, and pyrazinamide) for 2 months followed by daily isoniazid and rifampicin for an additional 4 months [10]. All enrolled patients accepted home-based the Directly Observed Treatment strategy, as it required less effort and a more convenient and flexible time schedule negotiated between the patient and volunteer. The regimens would be adjusted by the physicians according to adverse drug effects, and results of drug susceptibility testing.

After the beginning of the treatment, all enrolled TB patients would be followed up for 6 months. The time points of the follow-up were 2 weeks, 1 month, 2 months, 5 months, and 6 months after initiation of treatment. The patients who were lost to follow-up at any time point were also excluded from the study. The sputum microbiology (AFS and Mtb culture) was monitored at each time point in patients who still experienced expectoration. Radiology examination (CT scan) and other laboratory tests such as blood routine test and live function were simultaneously performed.

### 2.3. Assessment of TB Treatment Outcomes

The outcomes of TB treatment were classified into two categories based on the World Health Organization definition: successful (treatment completed, cured) and unsuccessful outcomes (treatment failure, default, and death) [10]. Patients who had a negative sputum smear and culture in the last month of treatment and on at least one previous occasion was defined as cured. Patients who completed treatment but who did not meet the criteria for classification as cured or a failure were defined as treatment completed. Patients who had a positive sputum smear or culture at 5 months or more after treatment initiation were defined as failed treatment. Patients whose treatment was interrupted for two consecutive months or more was defined as defaulted treatment. Patients who died for any reason during the course of treatment were defined as death. Patients who were transferred to another recording and reporting unit and for whom the treatment outcome was not known were excluded. In addition, whether the patients had risk factors which could affect the treatment duration, such as presence of cavitation on the initial chest radiograph, having no radiographic improvement after 2 months of treatment, and having a positive AFS after 2 months of treatment was also recorded.

### 2.4. Serial T-SPOT Assay and Calculation of TBAg/PHA Ratio

Peripheral blood samples were collected at different time points from patients before and after TB treatment. T-SPOT assay (Oxford Immunotec, Oxford, UK) was serially performed before treatment and at 2 weeks, 1 month, 2 months, 5 months, and 6 months after treatment, according to the manufacturer’s instructions. Briefly, the isolated peripheral blood mononuclear cells (2.5 × 10^5^) were added to 96-well plates precoated with anti-IFN-γ antibody. Four wells were used for each patient: medium well, PHA well, early secreted antigenic target 6 (ESAT-6), and culture filtrate protein 10 (CFP-10) wells. Plates were incubated for 16–20 h at 37 °C with 5% CO_2_ and developed using an anti-IFN-γ antibody conjugate and substrate to detect the presence of secreted IFN-γ. Spot-forming cells (sfc) were counted with an automated ELISPOT reader (CTL Analyzers, Cleveland, OH, USA). Positive and negative results were defined according to the manufacturer’s recommendations. Results were considered undetermined if the spot amounts in the positive control were <20 or if spot amounts in the negative control were >10. The ratios of (1) ESAT-6 sfc to PHA sfc, and (2) CFP-10 sfc to PHA sfc were calculated; the larger of these two values was defined as the TBAg/PHA ratio of each patient.

### 2.5. Statistical Analysis

The results are presented as mean ± standard deviation (SD). Differences between different time points were analyzed by paired Student’s *t*-test. Differences between patients with successful and unsuccessful treatment outcomes were analyzed by the Mann–Whitney U-test. The chi-square test was used for categorical data. To aid visualization, a smoothing spline was fitted to the values of different timepoints to summarize the overall trend. Receiver operating characteristic (ROC) analysis was performed to determine the best cut-off value, and the area under the curve (AUC) was identified. AUCs of different groups were compared by using the DeLong test. Spearman’s rank correlation test for non-parametric data was employed to analyze the relationship between two factors. Statistical significance was determined as *p* < 0.05. Statistical analyses were performed using GraphPad Prism 8.0 (San Diego, CA, USA).

## 3. Results

### 3.1. Patients’ Characteristics

**Figure 1:**
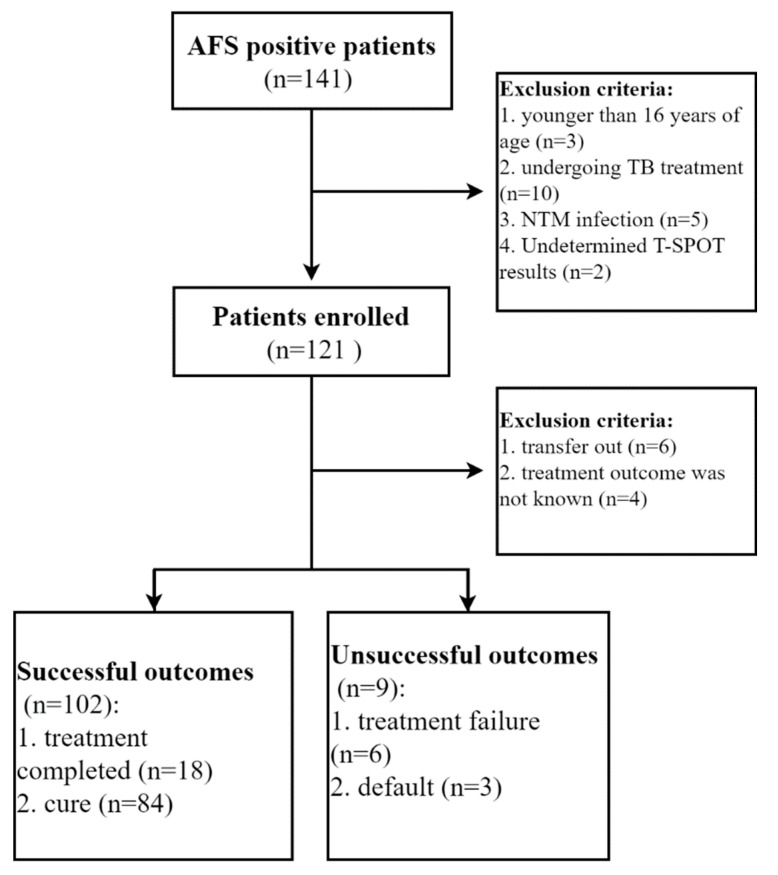
Flow diagram summarizing patient recruitment, exclusion criteria, and the patient groups.

There were 141 patients who had positive sputum AFS results in three centers between July 2018 and September 2018. After excluding 20 patients (younger than 16 years of age, *n* = 3; undergoing TB treatment, *n* = 10; NTM infection, *n* = 5; undetermined T-SPOT results, *n* = 2), a total of 121 patients were enrolled in this study. After excluding another 10 patients (transfer out, *n* = 6; without treatment outcome, *n* = 4), 111 patients were recorded with the final treatment outcome. Of them, 102 patients had successful treatment outcomes (treatment completed, *n* = 18; cure, *n* = 84), and 9 patients had unsuccessful treatment outcomes (treatment failure, *n* = 6; default, *n* = 3) (Figure 1). Among 6 patients with failed treatment outcomes, 2 were diagnosed as multidrug-resistance TB (isoniazid and rifampicin resistance). The demographic and clinical characteristics of the study participants in different outcome groups are shown in Table 1. No significant difference in age, gender, body mass index (BMI), blood pressure, routine laboratory indicators such as white blood cell (WBC), and lymphocyte count was observed between successful and unsuccessful treatment outcome groups.

### 3.2. T-SPOT Results in Patients with Successful Treatment Outcomes

**Figure 2:**
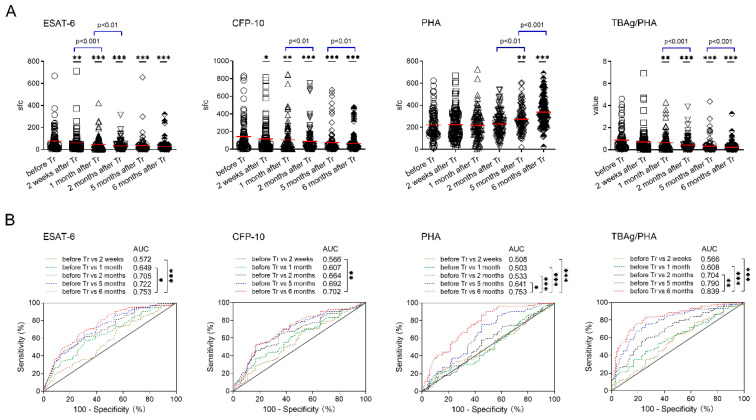
T-SPOT results in patients with successful treatment outcomes. (**A**) Dot plots showing ESAT-6 sfc, CFP-10 sfc, PHA sfc, and the TBAg/PHA ratio in patients before and after different time points (2 weeks, 1 month, 2 months, 5 months, 6 months) of TB treatment. Bars indicate means (* *p* < 0.05, ** *p* < 0.01, and *** *p* < 0.001, compared with before treatment). (**B**) Receiver operating characteristic analysis was performed for ESAT-6 sfc, CFP-10 sfc, PHA sfc, and the TBAg/PHA ratio to determine threshold values for distinguishing between before treatment and after different time points of treatment. * *p* < 0.05, ** *p* < 0.01, and *** *p* < 0.001. ESAT-6, early secreted antigenic target 6; CFP-10, culture filtrate protein 10; PHA, phytohemagglutinin; sfc, spot-forming cells. TBAg/PHA, the ratio of TB-specific antigen to phytohemagglutinin. Tr, treatment.

Both ESAT-6 and CFP-10 sfc in TB patients after 2 weeks of TB treatment were significantly decreased compared with those before treatment. Conversely, PHA sfc in TB patients after 5 months of anti-TB treatment was significantly increased compared with those before treatment, despite no obvious change in the first few months after initiation of treatment. Accordingly, the TBAg/PHA ratio in TB patients after 1 month of TB treatment was significantly decreased compared with those before treatment. We further compared T-SPOT results between adjacent time points, and found that ESAT-6 sfc displayed a more pronounced decrease in the early stage of treatment (within 2 months). In contrast, the decrease of CFP-10 sfc and the TBAg/PHA ratio was more pronounced in the late stage of treatment (after 2 months) (Figure 2A). Furthermore, we found that ESAT-6 sfc, CFP-10 sfc, and the TBAg/PHA ratio all showed a decreased trend after initiation of treatment. Conversely, PHA sfc showed an increased trend especially after 2 months of treatment. Nevertheless, a high standard deviation of both TBAg and PHA sfc supported the notion that T-SPOT results showed a large variation among TB patients, no matter before or after treatment (Figure 2A).

ROC analysis was performed to evaluate the differential effect of these indicators on successful treatment outcome. We found that T-SPOT related indicators, including ESAT-6 sfc, CFP-10 sfc, PHA sfc and the TBAg/PHA ratio, in patients after 6 months of treatment had an optimal effect on distinguishing between before and after successful treatment. However, most of these indicators had moderate performance in distinguishing between before and after successful treatment, and the AUC ranged from 0.702 to 0.839 (Figure 2B). Among these indicators, the TBAg/PHA ratio performed best. If using >0.193 as the cut-off value, the sensitivity and specificity of the TBAg/PHA ratio in distinguishing before and after 6 months of treatment were 74.51% and 82.35%, respectively (Table 2).

### 3.3. Classification Groups in Patients with Successful Treatment Outcomes

The patients with risk factors, such as presence of cavitation on the initial chest radiograph, having no radiographic improvement after 2 months of treatment, and having a positive AFS after 2 months of treatment, were reported to have prolonged treatment duration [11,12]. Thus, the patients with successful treatment outcomes were classified into a non-risk factor group (*n* = 72) and a risk factor group (*n* = 30; included 12 patients with presence of cavitation on the initial chest radiograph, 14 patients having no radiographic improvement after 2 months of treatment, and 4 patients having a positive AFS after 2 months of treatment).

**Figure 3:**
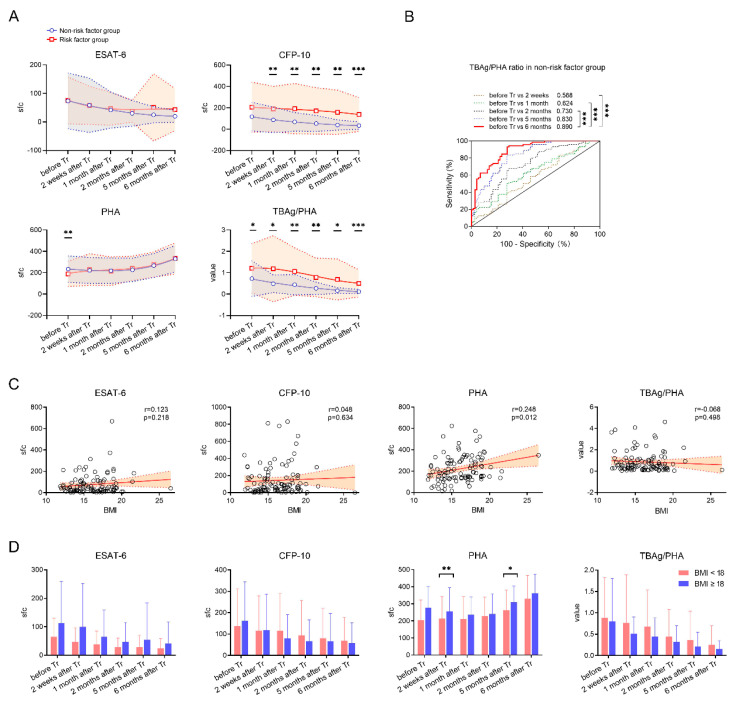
Classification groups in patients with successful treatment outcomes. (**A**) Dynamic analysis of the T-SPOT results in patients with and without risk factors after TB treatment. Data in each time point are expressed as mean ± SD (* *p* < 0.05, ** *p* < 0.01, and *** *p* < 0.001, between patients with and without risk factors in each time point). The smoothing cubic splines were fitted to the values of different parameters to summarize the overall trend. (**B**) Receiver operating characteristic analysis was performed for the TBAg/PHA ratio to determine threshold values for distinguishing between before treatment and after different time points of treatment. *** *p* < 0.001. (**C**) Correlation between T-SPOT results (ESAT−6 sfc, CFP−10 sfc, PHA sfc, and the TBAg/PHA ratio) and BMI in patients before treatment. (**D**) Bar graphs showing ESAT−6 sfc, CFP−10 sfc, PHA sfc, and the TBAg/PHA ratio in patients with BMI ≥ 18 and BMI < 18 at different time points after treatment. * *p* < 0.05, ** *p* < 0.01. ESAT−6, early secreted antigenic target 6; CFP−10, culture filtrate protein 10; PHA, phytohemagglutinin; sfc, spot-forming cells. TBAg/PHA, the ratio of TB-specific antigen to phytohemagglutinin. BMI, body mass index.

Similarly, ESAT-6 sfc, CFP-10 sfc, and the TBAg/PHA ratio all displayed a decreased trend but there was a progressive increase in PHA sfc, in both non-risk and risk factor groups after treatment. However, in most time points after treatment, patients with risk factors displayed significantly higher values of CFP-10 sfc and the TBAg/PHA ratio compared to those without a risk factor (Figure 3A). Notably, the TBAg/PHA ratio in patients without a risk factor was of important value in differentiation between before and after successful treatment. The optimal AUC of the TBAg/PHA ratio reached up to 0.890 (95% CI, 0.839–0.942). If using the cut-off value of >0.259, the sensitivity and specificity of the TBAg/PHA ratio in distinguishing before and after 6 months of treatment were 93.06% and 72.22%, respectively (Figure 3B).

Considering that duration of TB treatment is also prolonged in patients with weight loss [13], we further classified the patients into high (≥18) and low (<18) BMI groups. As expected, PHA sfc in TB patients before treatment was significantly positively correlated with BMI (Figure 3C). Accordingly, patients with BMI ≥ 18 showed significantly higher PHA sfc than those with BMI < 18 in some time points (2 weeks and 5 months) after treatment. Conversely, although the TBAg/PHA ratio trended higher in low BMI versus high BMI patients after treatment, the difference failed to achieve statistical significance (Figure 3D).

### 3.4. T-SPOT Results in Patients with Unsuccessful Treatment Outcomes

**Figure 4:**
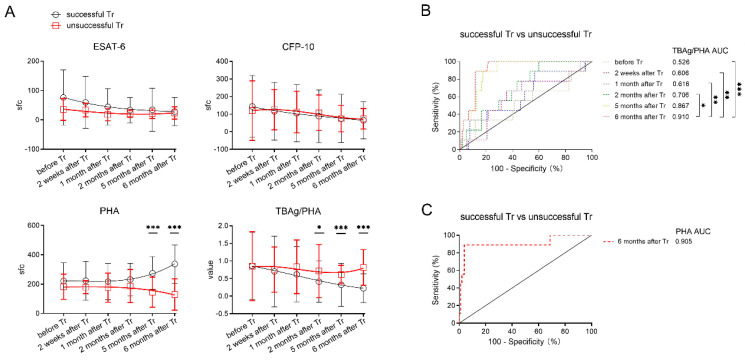
Comparing T-SPOT results between patients with successful and unsuccessful treatment outcomes. (**A**) Dynamic analysis of the T-SPOT results in patients with successful and unsuccessful treatment outcomes. Data in each time point are expressed as mean ± SD (* *p* < 0.05, *** *p* < 0.001, between patients with successful and unsuccessful treatment outcomes in each time points). The smoothing cubic splines were fitted to the values of different parameters to summarize the overall trend. (**B**) Receiver operating characteristic analysis was performed for the TBAg/PHA ratio and (**C**) PHA to determine threshold values for distinguishing between patients with successful and unsuccessful treatment outcomes (* *p* < 0.05, ** *p* < 0.01, *** *p* < 0.001). ESAT−6, early secreted antigenic target 6; CFP−10, culture filtrate protein 10; PHA, phytohemagglutinin; sfc, spot-forming cells. TBAg/PHA, the ratio of TB-specific antigen to phytohemagglutinin; Tr, treatment.

Notably, ESAT-6 sfc in patients with unsuccessful treatment outcomes did not show a decreased trend, and CFP-10 even showed a transient increase after initiation of treatment. In contrast to the gradual increase of PHA sfc in patients with successful treatment outcomes, PHA sfc in patients with unsuccessful treatment outcomes showed a progressive decrease after treatment. Accordingly, unlike successfully treated patients, patients with unsuccessful treatment outcomes showed persistently high levels of the TBAg/PHA ratio after treatment. Notably, patients with unsuccessful treatment outcomes displayed significantly higher levels of the TBAg/PHA ratio and lower levels of PHA compared to those with successful treatment outcomes (Figure 4A).

ROC analysis showed that the TBAg/PHA ratio in patients after 6 months of treatment showed a certain potential in distinguishing between patients with successful and unsuccessful treatment outcomes. The optimal AUCs of the TBAg/PHA ratio and PHA were 0.910 (95% CI, 0.850–0.970) and 0.905 (95% CI, 0.776–1), respectively (Figure 4B,C).

## 4. Discussion

The strategies for the monitoring of TB treatment remains a great challenge in clinical practice. Theoretically, the phenotype and function of Mtb-specific lymphocytes can be used for monitoring TB treatment, as they will be changed with the decrease of bacterial load. Consistent with this notion, the activation markers CD38, HLA-DR, and Ki-67 expressed on Mtb-specific CD4^+^ T cells in TB patients reverted to healthy or latent TB infection phenotype at the end of treatment, supporting the idea that phenotypic changes of Mtb-specific T cells are potential markers for monitoring treatment efficacy [14,15,16]. Similarly, after antigen stimulation, the secretion of Mtb-specific cytokines such as IFN-γ, TNF-α, and IL-2 are also the potential markers for monitoring TB treatment [17,18,19]. However, although the detection of Mtb-specific markers by flow cytometry is not affected by other diseases, these methods are limited by the need to analyze sufficient target events to be able to identify small subsets with high statistical significance. These methods are also complicated and need antigen stimulation in vitro. As a result, although the detection of Mtb-specific markers is a promising direction for monitoring TB treatment, these methods can be impractical in the clinical setting so far.

On the other side, some serum markers such as IP-10 and sCD14 were significantly decreased after TB treatment, indicating that these serum markers could serve as potential tools for monitoring therapy efficacy [20,21,22]. Conversely, the expression of perforin and granzyme B in CD8^+^ T lymphocytes was increased after TB treatment, supporting the idea that these intracellular markers might be also used for prediction of treatment outcomes of active TB [23]. Although these methods were more easily performed in clinical practice, the levels of these markers were also altered in many other inflammatory diseases, which leads to the low specificity of using these markers for monitoring TB treatment. On the other hand, the results of CT after TB treatment were mainly described as reduced pulmonary shadow or improved lesion. The patients who showed disappearance of abnormal shadow after treatment were rare. In addition, the interpretation of CT results had the difficulty of subjectivity. Thus, CT results also have limited value in monitoring TB treatment.

T-SPOT assay, as one of two commercially Interferon-gamma release assays, also detects the number of Mtb-specific lymphocytes [24,25,26]. Theoretically, the number of antigen-specific lymphocytes is correlated with the Mtb bacterial load, and will be decreased in active TB patients after treatment. Hereafter, T-SPOT assay might have potential in monitoring TB treatment. However, studies have shown that although the mean values of antigen sfc in T-SPOT assay show a downward trend during treatment, both qualitative and quantitative results of T-SPOT assay are only of limited value in monitoring TB treatment [2,27,28,29,30,31,32]. A large degree of variation in T-SPOT results in different patients, especially in those with immune suppression as discussed in our previous studies [6,33], might cause such disparity. Consistent with this notion, previous data have shown that some factors, such as age, being over-weight, longer periods of illness before hospitalization, and immunosuppressive therapy, may contribute to reduced or even negative T-SPOT results [34,35]. As a result, it is common to see TB patients with a low value of T-SPOT results in clinical practice, which leads to low T-SPOT results not correlating with successful treatment. Thus, the current evidence do not support using T-SPOT antigen results for monitoring TB treatment.

Consistent with this notion, we observed that although the T-SPOT results were significantly decreased even after 2 weeks of TB treatment, the direct application of T-SPOT antigen results was of limited value in predicting a successful treatment outcome. Differently, we found that the PHA results gradually increased after treatment, especially at 6 months of treatment. This is in accordance with previous studies indicating that host immunity is restored after TB treatment [36,37,38]. The lymphocyte response to PHA stimulation reflects the functional potential of them [39], and the increase of PHA results in T-SPOT assay indicates the restoration of lymphocyte function in TB patients after treatment. Thus, calculation of the TBAg/PHA ratio, which depends on the combination effect of the T-SPOT antigen and PHA results, is better than directly using T-SPOT antigen results in monitoring TB treatment. Generally, the essence of the TBAg/PHA ratio is that it can eliminate the individual immune variation on T-SPOT assay. 

The advantages of the TBAg/PHA ratio are summarized as follows: (1) the decrease of the TBAg/PHA ratio in patients at 6 months post treatment displays relatively high performance in identifying patients with successful treatment outcome, especially in those without risk factors; (2) the persistence of a high value of the TBAg/PHA ratio could be used as an indicator to early predict the unsuccessful outcome of treatment; and (3) T-SPOT assay is an independent method and has been widely used in clinical practice, and the method does not need to combine other laboratory tests or special equipment such as flow cytometry. Of note, the most limitation of the TBAg/PHA ratio is that it cannot be used in TB patients with negative T-SPOT results. Actually, there is a certain proportion of TB patients with negative T-SPOT results in clinical practice, especially in those with immunosuppression. Moreover, the high cost of reagents might preclude widespread use of T-SPOT assay in resource-limited settings.

## 5. Conclusions

This study confirms that although T-SPOT antigen results gradually decrease in active TB patients after treatment, they are of limited value in monitoring TB treatment because of large variation among individuals. Importantly, the restoration of PHA results after successful treatment in T-SPOT assay suggests a new role of PHA sfc in the treatment of TB. Accordingly, the TBAg/PHA ratio in T-SPOT assay shows persistently high levels in patients with unsuccessful treatment outcomes, which indicates the potential value of this ratio in the treatment monitoring of patients with TB.

## Figures and Tables

**Table 1:** Demographic and clinical characteristics of pulmonary TB patients in different outcome groups.

Characteristic		Successful Outcome (*n* = 102)	Unsuccessful Outcome (*n* = 9)	*p* Value
Mean age (mean ± SD), years		39.22 ± 15.28	44.89 ± 20.20	0.401
Male sex		70 (68.62)	6 (66.67)	0.903
Immunosuppressive conditions				
	HIV infection	2 (1.96)	0	
	malignancy	4 (3.92)	0	
	autoimmune disease receiving treatment	3 (2.94)	0	
	transplantation receiving treatment	1 (0.98)	0	
	diabetes	5 (4.90)	1 (11.11)	0.405
	chronic renal failure	4 (3.92)	0	
BMI		16.14 ± 2.507	16.42 ± 1.578	0.642
Blood pressure				
	systolic pressure	113.4 ± 15.65	108.1 ± 8.100	0.311
	diastolic pressure	71.79 ± 9.669	69.22 ± 7.823	0.531
WBC count (mean ± SD), ×10^9^/L		7.2 ± 2.008	7.034 ± 2.202	0.682
Lymphocyte count, ×10^9^/L		1.672 ± 0.569	1.611 ± 0.405	0.823

Data are presented as numbers (%) or mean ± SD. TB, tuberculosis; BMI, body mass index; WBC, white blood cells; SD, standard deviation.

**Table 2:** The optimal AUC of different indicators in T-SPOT results in differentiation between before and after successful treatment.

	Optimal AUC (95% CI)	Cutoff Value	Sensitivity % (95% CI)	Specificity % (95% CI)
ESAT-6	0.753 (0.687–0.819)	21.5	65.69 (55.63–74.81)	72.55 (62.82–80.92)
CFP-10	0.702 (0.630–0.774)	25.5	51.96 (41.84–61.96)	83.33 (74.66–89.98)
PHA	0.753 (0.687–0.820)	195.5	89.22 (81.52–94.49)	51.96 (41.84–61.96)
TBAg/PHA ratio	0.839 (0.785–0.894)	0.193	76.47 (67.04–84.31)	81.37 (72.45–88.40)

AUC, area under the curve; T-SPOT, T-SPOT.TB; ESAT-6, early secreted antigenic target 6; CFP-10, culture filtrate protein 10; PHA, phytohemagglutinin; TBAg/PHA, the ratio of TB-specific antigen to phytohemagglutinin.

## Data Availability

All data is included in the manuscript.
